# Quantitation of antibiotics in fresh fermentation medium by hydrophilic interaction chromatography mass spectrometry

**DOI:** 10.1007/s00216-025-05775-6

**Published:** 2025-02-21

**Authors:** Nadia Marcon, Mathias Rüdt, Joachim Klein, Saša M. Miladinović

**Affiliations:** 1 Institute of Life Sciences, School of Engineering, University of Applied Sciences West Switzerland, Sion, Switzerland; 2 Lonza AG, 3930 Visp, Switzerland

**Keywords:** HILIC-MS, Antibiotics, Fresh fermentation medium, Matrix effect, Solid-phase extraction

## Abstract

This study presents the development of a sophisticated liquid chromatography-mass spectrometry approach leveraging hydrophilic interaction chromatography (HILIC) for the quantification of kanamycin and spectinomycin in fermentation media. The method was validated per International Council for Harmonisation guidelines, demonstrating robust linearity, precision, and accuracy. To mitigate pronounced matrix effects common to complex fermentation matrices, sample preparation was thoroughly optimized with solid-phase extraction employing MCX sorbent, thereby enhancing recovery rates and minimizing analytical interference. The validated protocol demonstrated high correlation coefficients (*R* > 0.998), underscoring its robustness and reliability for the accurate quantification of antibiotics in challenging bioprocess environments, providing a valuable analytical tool for bioreactor system monitoring.

**Graphical Abstract:**

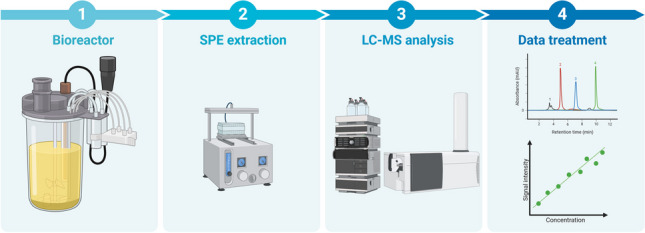

**Supplementary Information:**

The online version contains supplementary material available at 10.1007/s00216-025-05775-6.

## Introduction

Bioprocesses are central to modern processing industries for producing components that are expensive to obtain through classical chemical synthesis. These products range from small bulk chemicals like citric acid to complex specialty chemicals and large protein-based biopharmaceuticals [1]. Bioprocesses employ different organisms cultivated in suitable media within bioreactors to generate the desired products [2]. The medium plays a crucial role in providing the optimal conditions for growth and production, typically comprising carbon and nitrogen sources, trace elements, and other essential molecules. Often, components are added that are not chemically pure substances but complex mixtures, such as yeast extract, fetal bovine serum, or food processing by-products [1–3]. Consequently, biotechnological media are complex solutions containing numerous components.

Bioreactor fermentation is a cornerstone of industrial biotechnology, offering a controlled environment for microorganisms to achieve their biochemical potential [4]. This process involves the systematic cultivation of microorganisms in a closed system, which is essential for the large-scale production of pharmaceuticals, biofuels, enzymes, and other bioproducts [5]. The controlled conditions within a bioreactor—encompassing nutrient availability, temperature, pH, and aeration—are precisely optimized to exploit the metabolic activities of the chosen microorganisms, resulting in the efficient conversion of raw materials into valuable compounds [6]. Bioreactor fermentation not only facilitates the scalable synthesis of bioproducts but also provides a fertile ground for exploring microbial physiology and metabolic pathways, thereby advancing bioprocess optimization and sustainability.

The formulation of an appropriate fermentation medium is critical to the success of microbial cultivation and the subsequent production of the desired bioproducts [7]. The medium serves as a metabolic playground for microorganisms, supplying essential nutrients, carbon sources, and trace elements necessary for their growth and productivity. The complex balance of components, precisely tailored to the specific requirements of the target microorganism, is crucial for maximizing the efficiency and yield of the bioprocess. Developing an optimal fermentation medium demands a comprehensive understanding of microbial physiology, substrate utilization, and the interplay of environmental factors within the bioreactor. This exploration is fundamental to achieving reproducibility, scalability, and sustainability in bioreactor-based production systems [2].

Incorporating antibiotics into fermentation media for bioreactors is a strategic measure to create a controlled and sterile environment that promotes targeted microbial growth. Antibiotics, known for their antimicrobial properties, serve dual purposes in bioprocessing: they protect the fermentation process from potential contamination by undesirable microorganisms, ensuring culture purity, and they can also influence the yield and quality of the desired bioproduct. Selecting the appropriate antibiotics and determining their optimal concentrations are critical factors, striking a balance between inhibiting contaminants and preserving the vitality of the cultivated microorganisms. This integration of antibiotics into the fermentation medium represents a sophisticated approach to bioprocess optimization, where microbial dynamics and product formation are influenced by a tailored antimicrobial environment within the bioreactor [8]. Although antibiotics typically do not serve as nutrients, they simplify other manufacturing aspects by suppressing microbial growth in mammalian cell cultures [9], acting as selection agents to prevent plasmid loss in genetically modified organisms, or serving as the process’s target product [2]. In all cases mentioned, quantifying antibiotics in the medium is crucial, whether for measuring product titer, ensuring medium quality, or evaluating the effectiveness of subsequent wastewater treatment.

Liquid chromatography-mass spectrometry (LC–MS) is a powerful tool for identifying, quantifying, and characterizing antibiotics in complex matrices such as blood, serum, plasma, or tissue [10–12]. Hydrophilic interaction chromatography (HILIC) separation of polar antibiotics is more efficient than derivatization techniques [13, 14] and more compatible with mass spectrometry than ion-pair chromatography [15–17]. The synergy of HILIC separation and mass spectrometry detection (HILIC-MS) enables researchers to separate complex antibiotic mixtures, facilitating the qualitative and quantitative assessment of individual components in complex matrices [18, 19]. Such powerful analytical techniques are essential for understanding antibiotic dynamics in bioprocess monitoring and optimization and additionally evaluating the effectiveness of bioreactor-wastewater treatment.

However, there is a deficiency of information and publications addressing the LC–MS analysis of antibiotics in fermentation media for bioreactors, particularly concerning sample recovery and the influence of the unique matrix effect [11]. Notably, the literature lacks a focus on sample preparation and antibiotic extraction methods, leaving a significant gap in our understanding of these critical aspects within the context of bioprocessing [8, 18]. This study addresses this gap by providing one of the few validated methods specifically designed for this matrix. The findings not only contribute to a better understanding of matrix effects in bioprocess monitoring but also provide a foundation for future analytical advancements in industrial biotechnology.

## Experimental section

### Materials and reagents

Kanamycin, spectinomycin, hygromycin B, streptomycin, glacial acetic acid, 32% ammonia solution, mass spectrometry (MS)-grade formic acid, MS-grade 25% ammonia solution, glycerol, and lithium hydroxide were purchased from Merck (Darmstadt, Germany). Methanol and chromatographic grade acetonitrile were obtained from Avantor (Radnor, Pennsylvania), and MS-grade acetonitrile was sourced from Thermo Fisher Scientific (Massachusetts, USA). Yeast extract was procured from Biolife Italiana (Milan, Italy), and nutrient broth was acquired from Biokar Diagnostics (Pantin, France). The Oasis MCX 96-well plate (30 mg sorbent per well, 30 µm) was supplied by Waters (Baden-Dättwil, Switzerland). Ultra-purified water used in the experiments was produced from a Milli-Q purification system (Merck, Darmstadt, Germany).

### Preparation of the medium and standard solutions

Fresh fermentation medium was prepared by dissolving 0.6 g of glycerol, 5.0 g of nutrient broth, and 1.3 g of yeast extract in a 250 ml volumetric flask, which was then filled to volume with purified water. Stock solutions of kanamycin, spectinomycin, streptomycin, and hygromycin B were prepared at a concentration of 10 mg/ml in purified water, with streptomycin and hygromycin B serving as an internal standard (IS). These stock solutions were stored at − 20 °C in polypropylene tubes. A working solution of kanamycin (60 µg/ml) and spectinomycin (2 µg/ml) was prepared by diluting the stock solutions in fresh fermentation medium. This working solution was then further diluted stepwise in the medium to prepare the calibration standards, which ranged from 0.3 to 6.0 µg/ml for kanamycin and 10 to 200 ng/ml for spectinomycin. Each calibration standard was spiked with either streptomycin or hygromycin B at concentrations of 0.8 µg/ml and 0.5 µg/ml, respectively.

### Solid-phase extraction

Solid-phase extraction (SPE) plates were used for the purification and concentration of antibiotics before LC–MS analysis. The elution process was optimized to enhance sensitivity and specificity. The method used to purify the standards from fresh fermentation medium was as follows: the MCX well plate was conditioned with 1 ml of 1 M lithium hydroxide and 1.5 ml of purified water. Next, 500 µl of the standard was acidified by adding 50 µl of glacial acetic acid, and the resulting 550 µl acidified standard was loaded onto the well. The SPE wells were washed with 1.5 ml of 2% acetic acid in water, followed by 1.5 ml of acetonitrile. The target compounds were then eluted with 500 µl of 6% ammonia in methanol, transferred into polypropylene vials, and analyzed by the LC–MS system.

### Liquid chromatography-mass spectrometry

LC–MS analysis was performed using an Agilent 1260 Infinity II HPLC system (Agilent, Santa Clara, USA) coupled with an Agilent iFunnel 6550B quadrupole time-of-flight (qTOF) mass spectrometer equipped with an Agilent Jet Stream electrospray ionization (AJS ESI) source. Chromatographic separation was achieved on a Waters Atlantis Premier BEH Z-HILIC column (2.5 µm, 100 mm × 2.1 mm). The column oven was maintained at 50 °C. The mobile phases consisted of solvent A (20 mM ammonium formate, pH 3.0) and solvent B (0.1% formic acid in acetonitrile) [20]. The gradient elution program started with 10% mobile phase A, with a nonlinear gradient applied over 12 min to 85% mobile phase A. A sample volume of 10 µl was injected onto the column, with a solvent flow rate of 0.3 ml/min. A post-run time of 10 min was used to recondition the column. The gradient profile is provided in Supplementary Table 1.

High-resolution mass spectrometry in MS1 mode was employed to measure the mass-to-charge ratios (*m*/*z*) of the analyte ions. Mass spectrometry parameters were optimized for detecting the target antibiotic ions. The AJS ESI source operated with a capillary voltage of 3000 V, nozzle voltage of 1000 V, drying gas temperature of 200 °C at a flow rate of 14 l/min, nebulizing gas pressure of 20 psig, and a sheath gas temperature of 325 °C at a flow rate of 12 l/min. MS data were acquired in positive ion mode over the *m*/*z* range of 100–3200 in the standard mass range at 4 GHz high-resolution mode from 0 to 12 min. The octupole RF voltage was set at 750 V.

The data were processed using MassHunter Qualitative Analysis 10.0 software. Extracted ion chromatograms (EICs) were generated for spectinomycin, kanamycin, streptomycin (IS), and hygromycin B (IS); smoothed with a Gaussian function; and integrated. The extracted ion for spectinomycin was [M + H + H_2_O]^+^ at *m*/*z* 351.2; the [M + H]^+^ ion at *m*/*z* 333.2 was excluded due to matrix interference. The extracted ions for kanamycin, streptomycin, and hygromycin B were [M + H]^+^ at *m*/*z* 485.2, 582.3, and 528.2, respectively. The methodology using MS1 to collect the data serves as a screening tool with the potential to facilitate the non-targeted analysis of a diverse array of antibiotics [21, 22].

## Results and discussion

The developed LC–MS method was validated by assessing robustness, linearity, matrix effect, recovery, precision, and accuracy for the targeted antibiotics in the fermentation medium. Sample preparation and LC–MS analysis were validated according to International Council for Harmonisation (ICH) M10 guidelines. Initially, streptomycin was used as an internal standard. However, after observing poor streptomycin SPE recovery, hygromycin B was adopted as the internal standard.

### Linearity

**Fig. 1:**
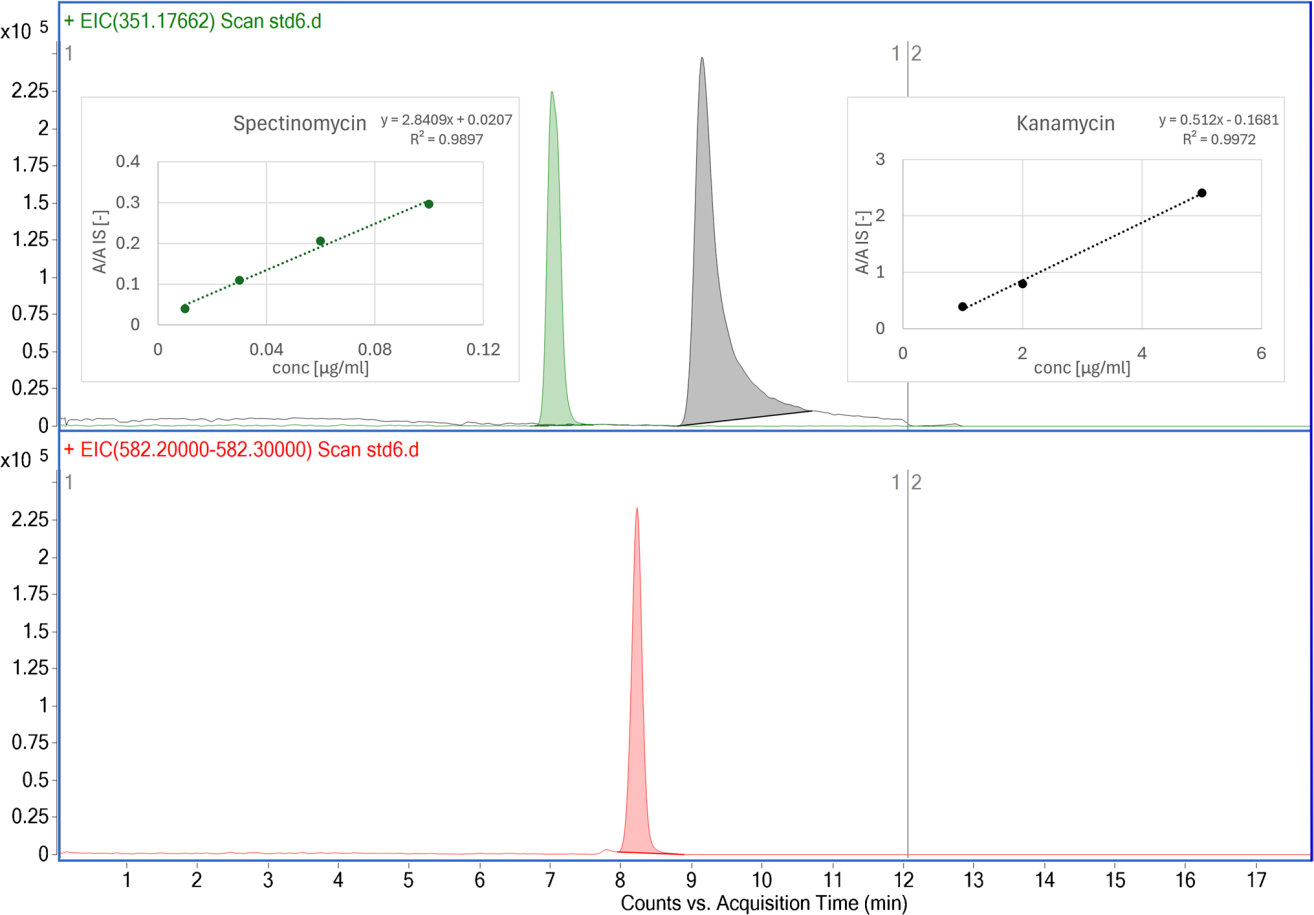
Upper panel: EICs of the analytes spectinomycin (green) and kanamycin (black). Lower panel: EIC of the internal standard streptomycin (pink). Insets (upper panel): calibration curves for spectinomycin (left) and kanamycin (right)

After optimizing the HILIC and MS conditions for the analytes, method development for quantifying spectinomycin and kanamycin continued with the establishment of a calibration curve in an aqueous matrix. Calibration standards for spectinomycin were prepared at concentrations of 0.01 µg/ml, 0.03 µg/ml, 0.06 µg/ml, and 0.1 µg/ml, while kanamycin standards were set at 1 µg/ml, 2 µg/ml, and 5 µg/ml. Streptomycin, initially used as an internal standard, was consistently maintained at 0.8 µg/ml throughout the analysis. The internal standard was selected because it elutes with similar retention times under HILIC separation. Its similar elution and ionization efficiency help to compensate for potential matrix effects. [23]. The initial calibration curve established in pure water showed excellent linearity and a good limit of quantitation, as depicted in Fig. 1.

### Matrix effect

**Fig. 2:**
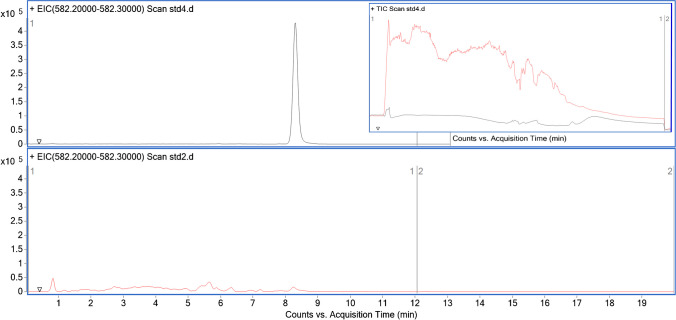
Extracted ion chromatograms of streptomycin in water (upper panel) and in fresh fermentation medium (lower panel). The inset displays high-resolution MS1 chromatograms of blank fresh fermentation medium (red trace) and blank water (black trace)

The standards were spiked into a fresh fermentation medium derived from bioreactor production to simulate real sample conditions. The calibration curve constructed using matrix-matched calibration standards was utilized for quantifying samples from the bioreactor. However, this standard prepared in fresh fermentation medium revealed a significant matrix effect, characterized by the suppression of the signal for both antibiotics and the internal standard. The lower chromatogram of Fig. 2 shows the high-resolution MS1 extracted ion chromatogram of the internal standard in fresh fermentation medium, while the upper chromatogram shows the EIC from pure water. The inset shows the total ion chromatogram (TIC) of the pure medium and pure water, illustrating the extent of the matrix effect. Matrix effects are common analytical challenges often resulting from complex sample compositions that affect the ionization efficiency of the target analytes [24]. While matrix effects in LC–MS and HILIC-MS are well documented, few studies address this issue in quantifying antibiotics in bioreactor media.

The persistent matrix effect necessitated further optimization of the HILIC-MS method and sample preparation parameters [20]. It was observed that direct sample injection by HILIC-MS was affected by the matrix effect without prior sample pretreatment. This indicated that rigorous method development and validation in the presence of complex matrices are necessary for accurate quantitative analysis. Enhanced sample preparation techniques, such as SPE, were considered to reduce the matrix components responsible for signal suppression. Implementing these corrective measures aimed to improve the accuracy and reliability of the quantitative determination.

The SPE cleanup and recovery using strong cation exchange (MCX) sorbent to enhance sample purity was evaluated [25]. A comparative analysis of six distinct SPE methodologies was conducted to identify the optimal approach for maximizing analyte recovery while minimizing matrix effects. The SPE sample preparation steps for each method are presented in Supplementary Table 2. Overlays of extracted ion chromatograms from fresh fermentation medium and water for each method are shown in Supplementary Figs. 1–6.

Method 5, presented in Supplementary Table 2, emerged as the superior method due to its enhanced recovery rates and low matrix effect. This efficacy is attributed to the significant reduction of matrix interferences, a common challenge in complex biological samples. Optimization of SPE conditions, including the pH and ionic strength of the washing and elution solvents, was important to achieving high recovery. The pH was measured and adjusted for aqueous buffers only. During the first washing step, a low pH facilitated the retention of the analytes on the sorbent while removing hydrophilic interfering compounds from the matrix. The second washing step, using 100% acetonitrile, effectively removed weak acids, neutrals, and hydrophobic interferences. The third, elution, step at high pH in an organic phase released spectinomycin and kanamycin from the sorbent. The robustness of the method was assessed through repeated analyses, screening a total of 56 runs, including pretests and validation parameters such as linearity, precision, accuracy, and SPE recovery. The method demonstrated a consistent mean recovery of 100%, with a relative standard deviation (RSD) of 7% across 25 results. The strategic selection of MCX sorbent, known for its strong cation exchange mixed with reversed-phase properties, was instrumental in the selective retention and elution of the target antibiotics.

This method’s implementation ensures reliable quantification of spectinomycin and kanamycin in fermentation media, providing a foundation for accurate dosing and monitoring of these antibiotics in bioreactor systems. The precision and accuracy achieved through this SPE technique highlights its potential application in routine quality control and evaluating the effectiveness of bioreactor-wastewater treatment.

The initial method validation using streptomycin as the internal standard revealed excellent linearity and a good limit of quantitation in a pure aqueous matrix. However, a significant matrix effect, characterized by signal suppression, was observed when the calibration standards were prepared in fresh fermentation medium. This highlighted the complexity of the bioreactor-derived samples and underscored the necessity of using matrix-matched calibration standards for accurate quantification.

Although method 5 successfully recovered spectinomycin and kanamycin, it failed to recover streptomycin effectively. Consequently, hygromycin B was selected as an alternative IS suitable for MCX SPE extraction. Hygromycin B was selected due to its good recovery, stable ionization, compatibility with the optimized SPE procedure, and similar retention behavior. The adoption of hygromycin B as an IS improved the method’s robustness, enabling accurate quantification of the target antibiotics in the complex fermentation medium. Overlays of extracted ion chromatograms from fresh fermentation medium and elution solution for method 5 with hygromycin B are shown in Supplementary Fig. 7.

#### Linearity in matrix

**Fig. 3:**
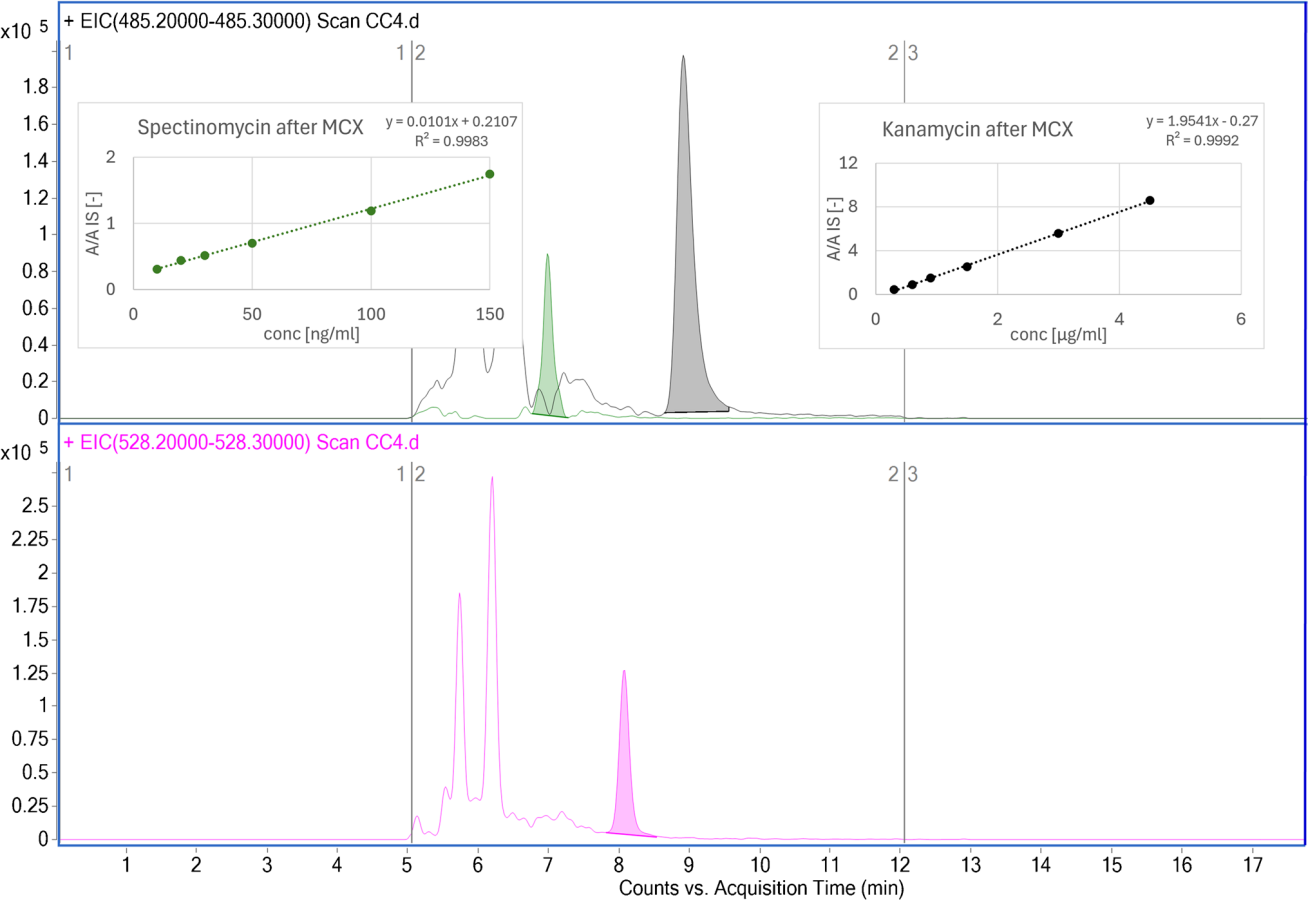
Upper panel: EICs of the analytes spectinomycin (green) and kanamycin (black). Lower panel: EIC of the internal standard hygromycin B (pink). Insets (upper panel): calibration curves for spectinomycin (left) and kanamycin (right) following the MCX cleanup of the samples

The calibration curves were established in the concentration range of limit of quantitation (LOQ) to 20-fold LOQ and demonstrated good linearity. The correlation coefficients (*r*^2^) were above 0.998 for both tested aminoglycosides in both matrix and elution solutions. The calibration curve in the fresh fermentation medium was constructed in the concentration range of 0.3 to 4.5 µg/ml for kanamycin and 10 to 150 ng/ml for spectinomycin, showing excellent linearity and a low LOQ (Fig. 3). These findings underscore the method’s applicability in monitoring antibiotic levels in bioreactor systems and wastewater.

Matrix effects were calculated by comparing the response of the target analytes spiked in the medium or elution solution at concentrations ranging from LOQ to 20-fold LOQ. For both analytes, a mean negative matrix effect (signal suppression) of − 4% for kanamycin and − 55% for spectinomycin was observed. The matrix effects were relatively consistent over the range of tested concentrations. This consistency indicates that while matrix effects are unavoidable, their impact can be managed through careful method optimization. Consequently, the high matrix effect for spectinomycin influenced the increase of the LOQ for the assay.

The high matrix effects, particularly for spectinomycin, pose a significant challenge despite the optimization of SPE conditions. While the current approach using MCX sorbent provided satisfactory recovery for kanamycin, the high level of signal suppression for spectinomycin suggests that additional strategies, such as the use of mixed-mode SPE, should be explored. Comparative studies evaluating these alternative approaches could provide deeper insights into mitigating matrix-related issues in complex fermentation media.

#### Precision

The method’s precision was assessed by six-time injecting (*n* = 6) a standard spiked in the matrix at a concentration of fivefold LOQ. The RSD was 0.8% for kanamycin and 1.9% for spectinomycin. The results are shown in Table 1. These metrics confirm the method’s reliability for routine quantitative analysis of antibiotics in fermentation media.

**Table 1:** Assessment of precision

Analyte	Concentration (ng/ml)	Precision (*n* = 6)	
		Mean relative peak area (SD)	RSD (%)
Spectinomycin	50	1.32 (0.02)	1.89
Kanamycin	1500	2.24 (0.02)	0.80

Accuracy was evaluated at three concentration levels (twofold LOQ, fivefold LOQ, and 15-fold LOQ) with three replicates each (*n* = 3). Kanamycin and spectinomycin were spiked in the matrix, and the full sample preparation steps were performed. The mean accuracy ranged between 88 and 106% as presented in Table 2, which is consistent with ICH guidelines.

**Table 2:** Assessment of accuracy

Analyte	Concentration (ng/ml)	Accuracy (*n* = 3)	
		Mean recovery (% (SD))	RSD (%)
Spectinomycin	20	94.07 (9.21)	9.80
	50	87.80 (6.74)	7.67
	150	88.18 (3.59)	4.07
Kanamycin	600	105.54 (2.84)	2.69
	1500	94.47 (0.97)	1.03
	4500	99.78 (1.43)	1.44

### SPE recovery

The recovery of the SPE cleanup was assessed at three concentration levels (twofold LOQ, fivefold LOQ, and 15-fold LOQ), each with three replicates (*n* = 3). Kanamycin and spectinomycin were spiked into the matrix and subjected to the SPE procedure. For recovery measurement, a blank matrix was also subjected to the SPE procedure, followed by spiking with kanamycin and spectinomycin in the purified matrix. Recovery ranged from 111 to 119% for kanamycin, indicating good analyte recovery. In contrast, spectinomycin recovery ranged from 37 to 76%, with higher concentrations yielding lower recovery. This suggests that the sorbent capacity may have been exceeded, highlighting the need to optimize sample load for accurate quantification. The RSDs of the replicates ranged from 2.3 to 5.6%. The results are collected in Table 3. An overlay of extracted ion chromatograms of SPE recovery of spectinomycin and kanamycin is shown in Supplementary Fig. 8.

**Table 3:** Assessment of SPE recovery

Analyte	Concentration (ng/ml)	SPE recovery (*n* = 3)	
		Mean recovery (% (SD))	RSD (%)
Spectinomycin	20	76.23 (4.26)	5.59
	50	49.55 (1.44)	2.90
	150	37.33 (0.99)	2.64
Kanamycin	600	111.50 (5.22)	4.68
	1500	111.40 (1.90)	1.70
	4500	119.05 (1.25)	1.05

Matrix effects are well-documented challenges in LC–MS analyses, often arising from the complex nature of biological matrices. In this study, the matrix effect necessitated further optimization of both the HILIC-MS method and the sample preparation process. Direct sample injection without prior sample pretreatment was inadequate, stressing the need for rigorous method development when working with complex matrices. Various SPE methods were evaluated to mitigate the matrix effect. The comparative analysis identified method 5, employing MCX sorbent, as the optimal approach.

Although the developed method was tailored for spectinomycin and kanamycin, findings suggest that the method may require further optimization or customization when applied to a broader range of antibiotic classes. Future research should focus on refining the SPE conditions or developing alternative cleanup strategies that can accommodate a wider spectrum of antibiotics, ensuring comprehensive applicability in bioprocess monitoring and control.

## Conclusion

This study highlights the development and validation of an LC–MS method utilizing HILIC for the quantification of spectinomycin and kanamycin in complex fermentation media. The optimized method, incorporating SPE using MCX sorbent, successfully addresses many challenges associated with matrix effects in such complex biological samples. While significant signal suppression was observed, especially for spectinomycin, careful method optimization allowed for consistent quantification within acceptable limits.

Using MS1 to collect data functions as a screening tool with the potential to facilitate the non-targeted analysis of a diverse array of antibiotics. This approach is valuable for ensuring medium quality and evaluating the effectiveness of subsequent wastewater treatments. The findings from this study not only enhance the reliability of spectinomycin and kanamycin quantification but also contribute to the broader field of untargeted antibiotic analysis. The methodological advancements presented here pave the way for more efficient and effective sample preparation techniques in LC–MS analytics.

However, the study also stresses the fundamental difficulties of analyzing highly polar antibiotics in such complex matrices, particularly when applying the method to a broader range of analytes. The findings highlight the need for continuous optimization and tailored approaches when quantifying antibiotics in bioprocess media, particularly where matrix effects are substantial.

Further research should explore alternative sample preparation strategies to reduce matrix interferences more effectively, potentially broadening the method’s applicability to other antibiotics frequently encountered in fermentation processes. The successful application of this method for quantifying spectinomycin and kanamycin provides a foundation for routine bioprocess monitoring and the analysis of fermentation media in industrial biotechnology.

## Supplementary Information

Below is the link to the electronic supplementary material.

Supplementary file1 (DOCX 3.36 MB)
