# Supplementary material for: Quantitation of antibiotics in fresh fermentation medium by hydrophilic interaction chromatography mass spectrometry
Source: Anal Bioanal Chem. 2025 Feb 21;417(9):1927–34. doi: 10.1007/s00216-025-05775-6 (PMC11914338; doi:10.1007/s00216-025-05775-6)
Supplement: Supplementary file 1 — Supplementary file1 (DOCX 3.36 MB) [file 216_2025_5775_MOESM1_ESM.docx]

Supplementary material

Quantitation of antibiotics in fresh fermentation medium by hydrophilic interaction chromatography mass spectrometry

Nadia Marcon, Mathias Rüdt, Joachim Klein†, Saša M. Miladinović*

Table S1. LC gradient program

| Time   [min] | Mobile phase A - 20 mM ammonium formate, pH 3.0  [%] | Mobile phase B - 0.1% formic acid in acetonitrile [%] |
| --- | --- | --- |
| 0.00 | 10.00 | 90.00 |
| 5.00 | 75.00 | 25.00 |
| 12.00 | 85.00 | 15.00 |
| 20.00 | 85.00 | 15.00 |

Table S2. Solid phase extraction steps for all the methods tested

The following solid phase extraction methods were developed on Oasis ®MCX 3 cc (60 mg) cartridges. If using other cartridge sizes, the volumes used at each method step must be adapted accordingly.

| Step | Method 1^1^ | Method 2^2^ | Method 3^2^ | Method 4^2^ | Method 5^2^ | Method 6^3^ |
| --- | --- | --- | --- | --- | --- | --- |
| Sample pretreatment  (1000 µl sample) | pH to 5.5 ± 0.5 | 100 µl glacial CH_3_COOH | 100 µl glacial CH_3_COOH | 100 µl glacial CH_3_COOH | 100 µl glacial CH_3_COOH | 1000 µl 4% H_3_PO_4_ |
| Conditioning | 3 ml MeOH  3 ml 2% CH_3_COOH | 2 ml LiOH 1M  3 ml water | 2 ml LiOH 1M  3 ml water | 2 ml LiOH 1M  3 ml water | 2 ml LiOH 1M  3 ml water | N/A |
| Loading | 1000 µl sample | 1100 µl sample | 1100 µl sample | 1100 µl sample | 1100 µl sample | 2000 µl sample |
| Washing | 3 ml 2% CH_3_COOH  3 ml water | 2.5 ml water  2.5 ml ACN | 2.5 ml water  2.5 ml ACN | 2.5 ml water  2.5 ml ACN | 3 ml 2% CH_3_COOH  3 ml ACN | 3 ml 2% CH_3_COOH  3 ml MeOH |
| Eluting | 1000 µl 20 % NH_4_OH in MeOH | 1000 µl 1M ammonium formate pH 9.5 | 1000 µl 20 % NH_4_OH in MeOH | 1000 µl 6 % NH_4_OH in MeOH | 1000 µl 6 % NH_4_OH in MeOH | 1000 µl 6 % NH_4_OH in MeOH |
| Sample post treatment | Evaporation + resuspention in 1000 µl water | N/A | N/A | N/A | N/A | N/A |


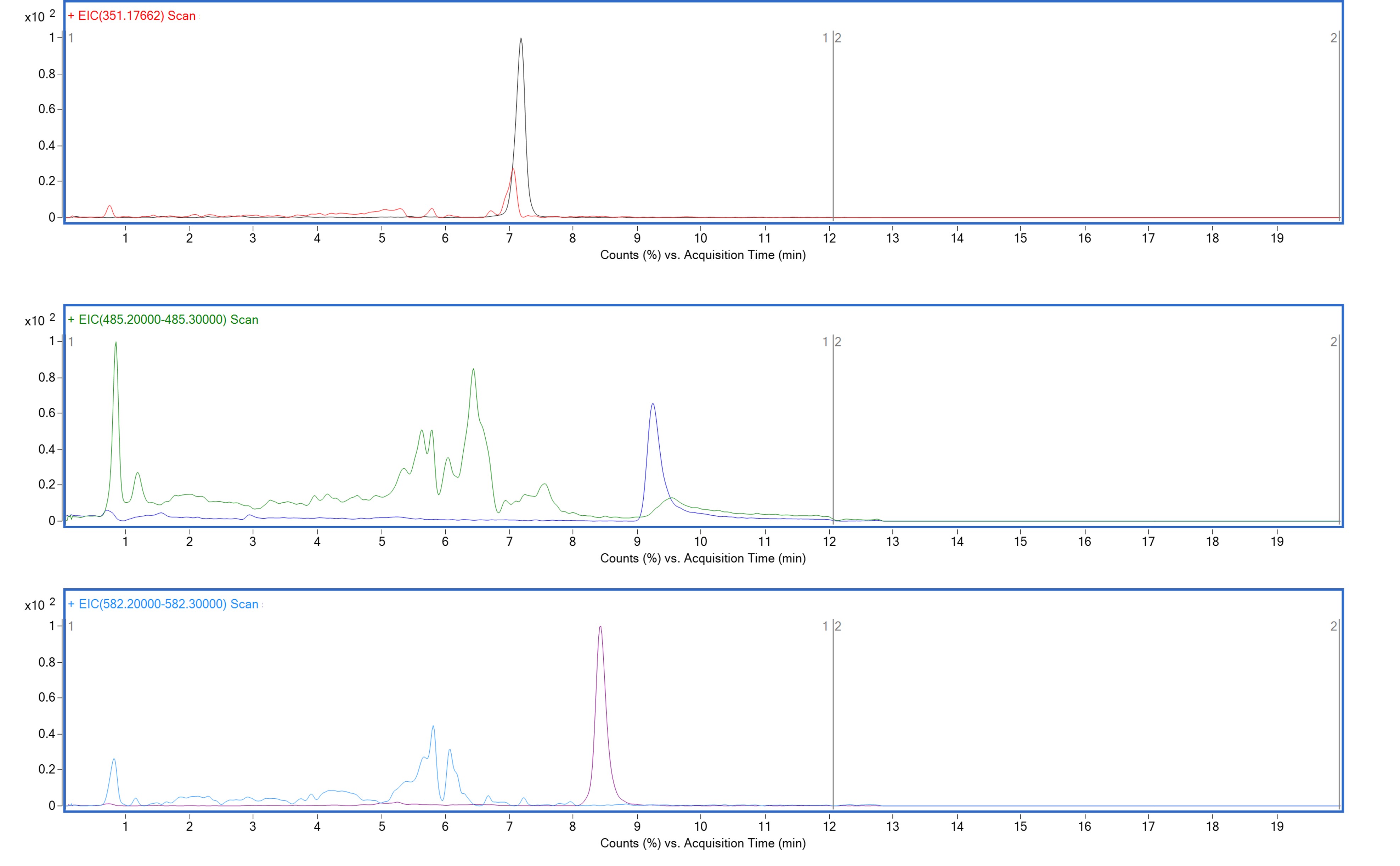


Figure S1. Overlays of extracted ion chromatograms after **Method 1** SPE clean-up of: Spectonomycin (upper) in fresh fermentation medium (red) and water (black); Kanamycin (middle) in fresh fermentation medium (green) and water (blue); Streptomycin (lower) in fresh fermentation medium (blue) and water (purple)


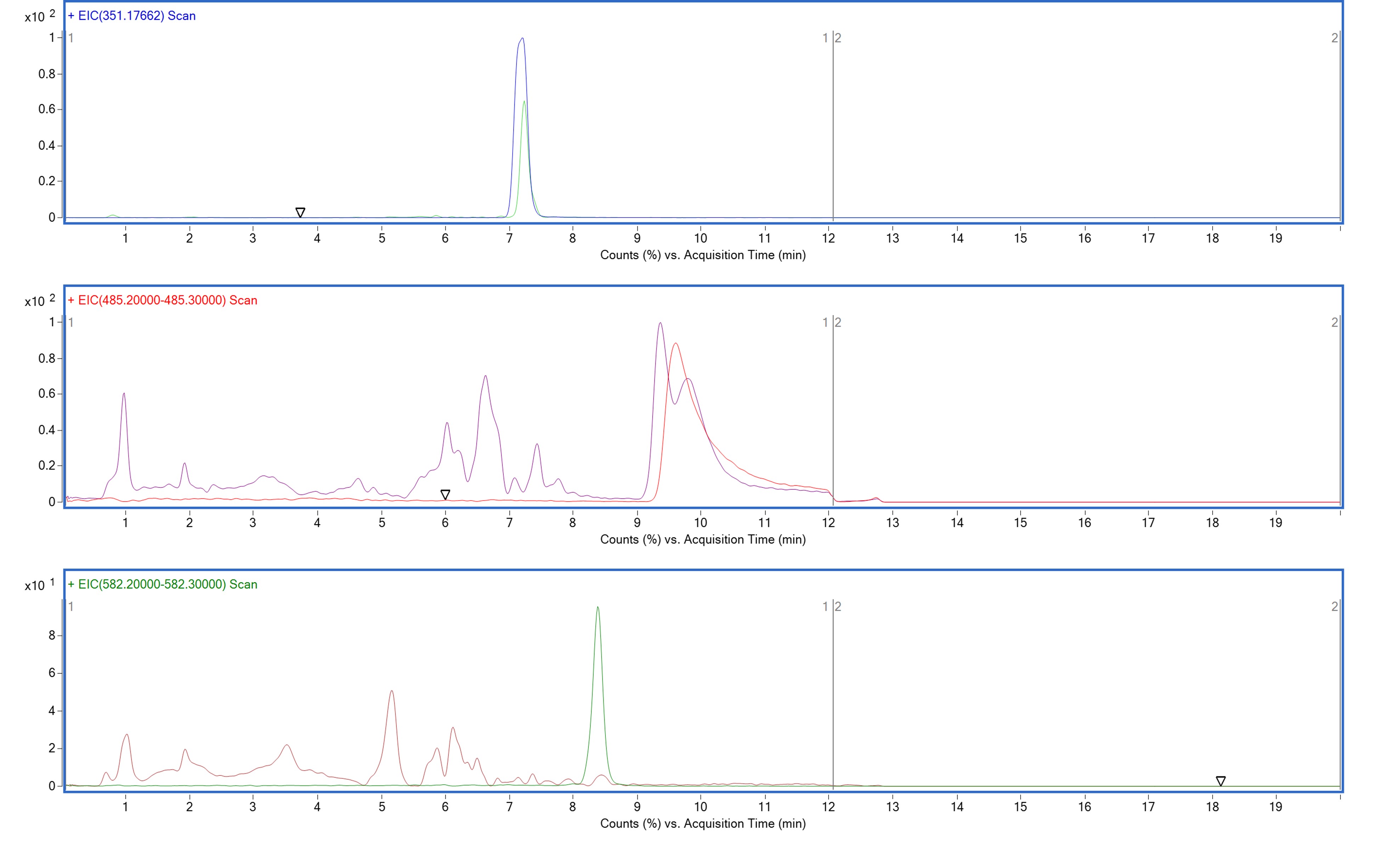


Figure S2. Overlays of extracted ion chromatograms after **Method 2** SPE clean-up of: Spectonomycin (upper) in fresh fermentation medium (green) and water (blue); Kanamycin (middle) in fresh fermentation medium (purple) and water (red); Streptomycin (lower) in fresh fermentation medium (brown) and water (green)


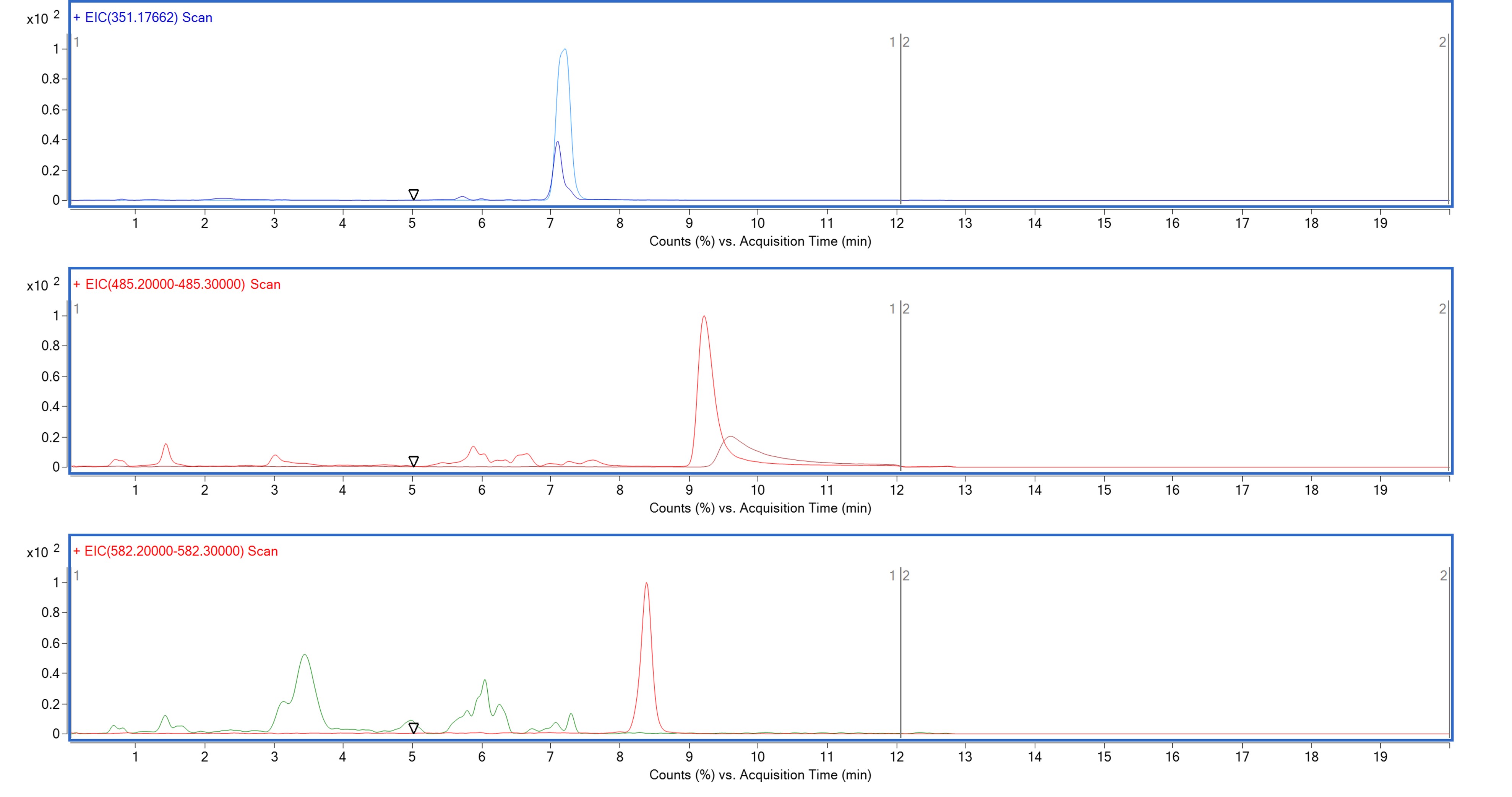


Figure S3. Overlays of extracted ion chromatograms after **Method 3** SPE clean-up of: Spectonomycin (upper) in fresh fermentation medium (dark blue) and water (light blue); Kanamycin (middle) in fresh fermentation medium (red) and water (brown); Streptomycin (lower) in fresh fermentation medium (green) and water (red)


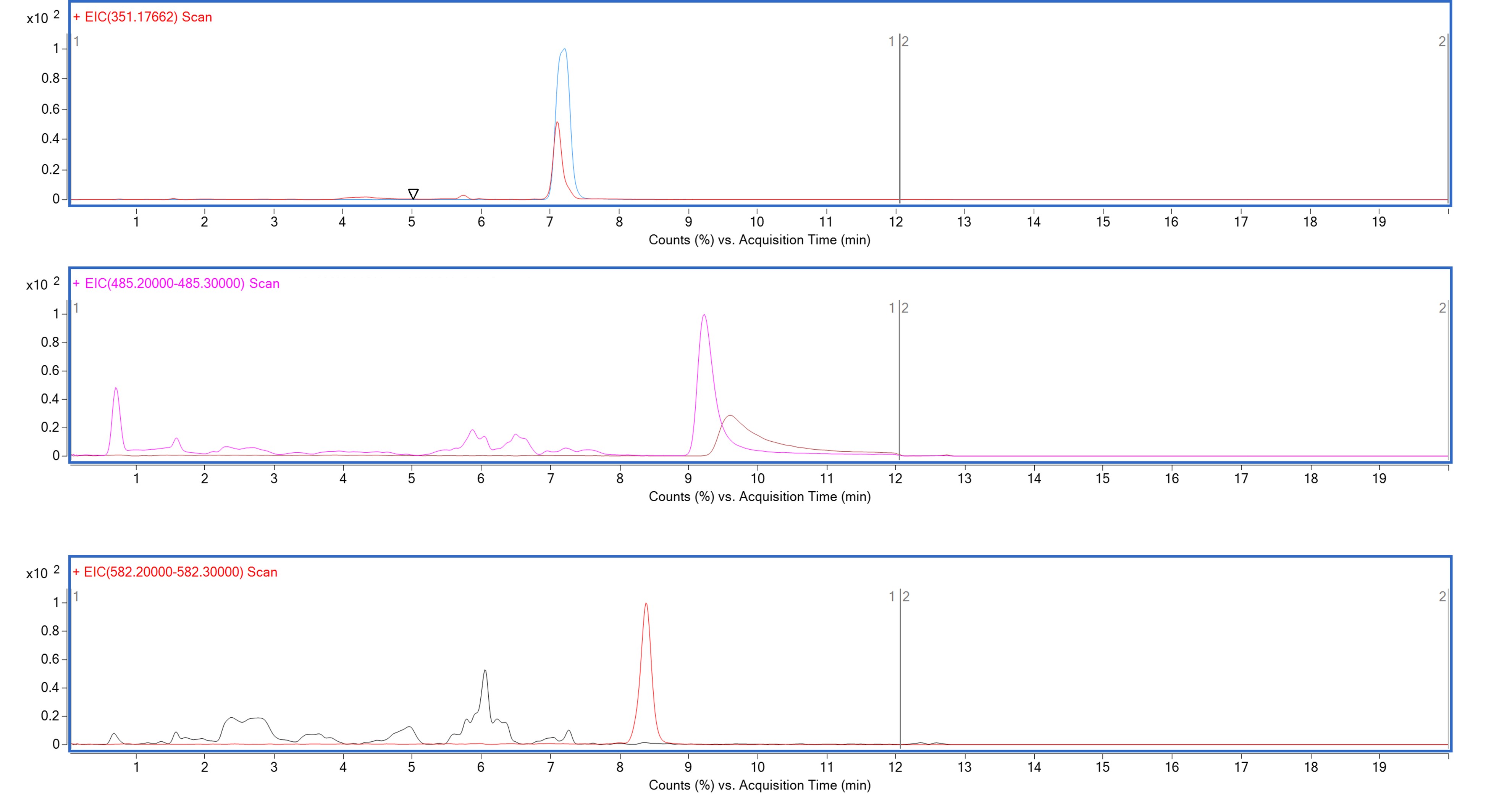


Figure S4. Overlays of extracted ion chromatograms after **Method 4** SPE clean-up of: Spectonomycin (upper) in fresh fermentation medium (red) and water (light blue); Kanamycin (middle) in fresh fermentation medium (pink) and water (brown); Streptomycin (lower) in fresh fermentation medium (black) and water (red)


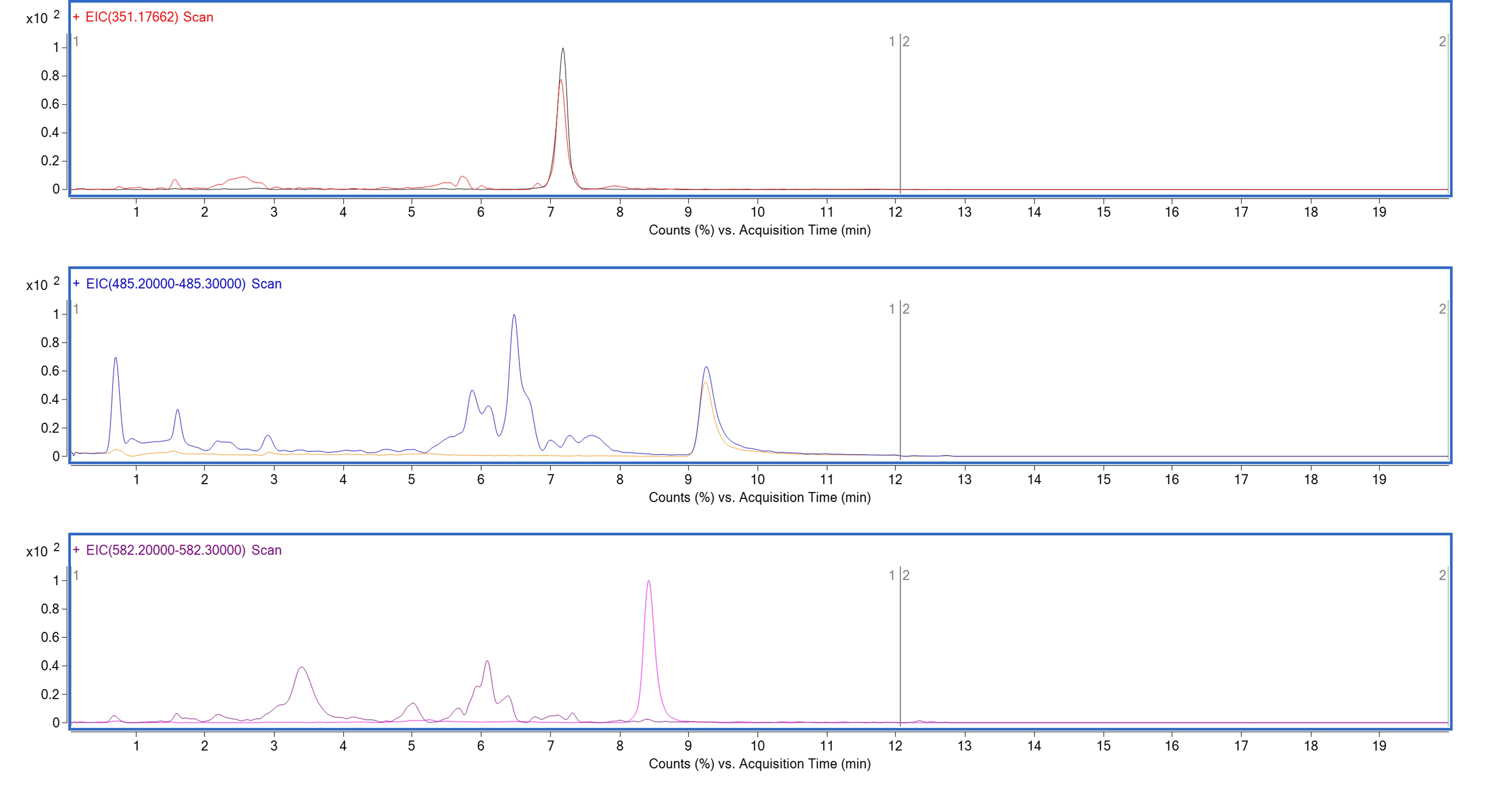


Figure S5. Overlays of extracted ion chromatograms after **Method 5** SPE clean-up of: Spectonomycin (upper) in fresh fermentation medium (red) and water (black); Kanamycin (middle) in fresh fermentation medium (blue) and water (yellow); Streptomycin (lower) in fresh fermentation medium (purple) and water (pink)


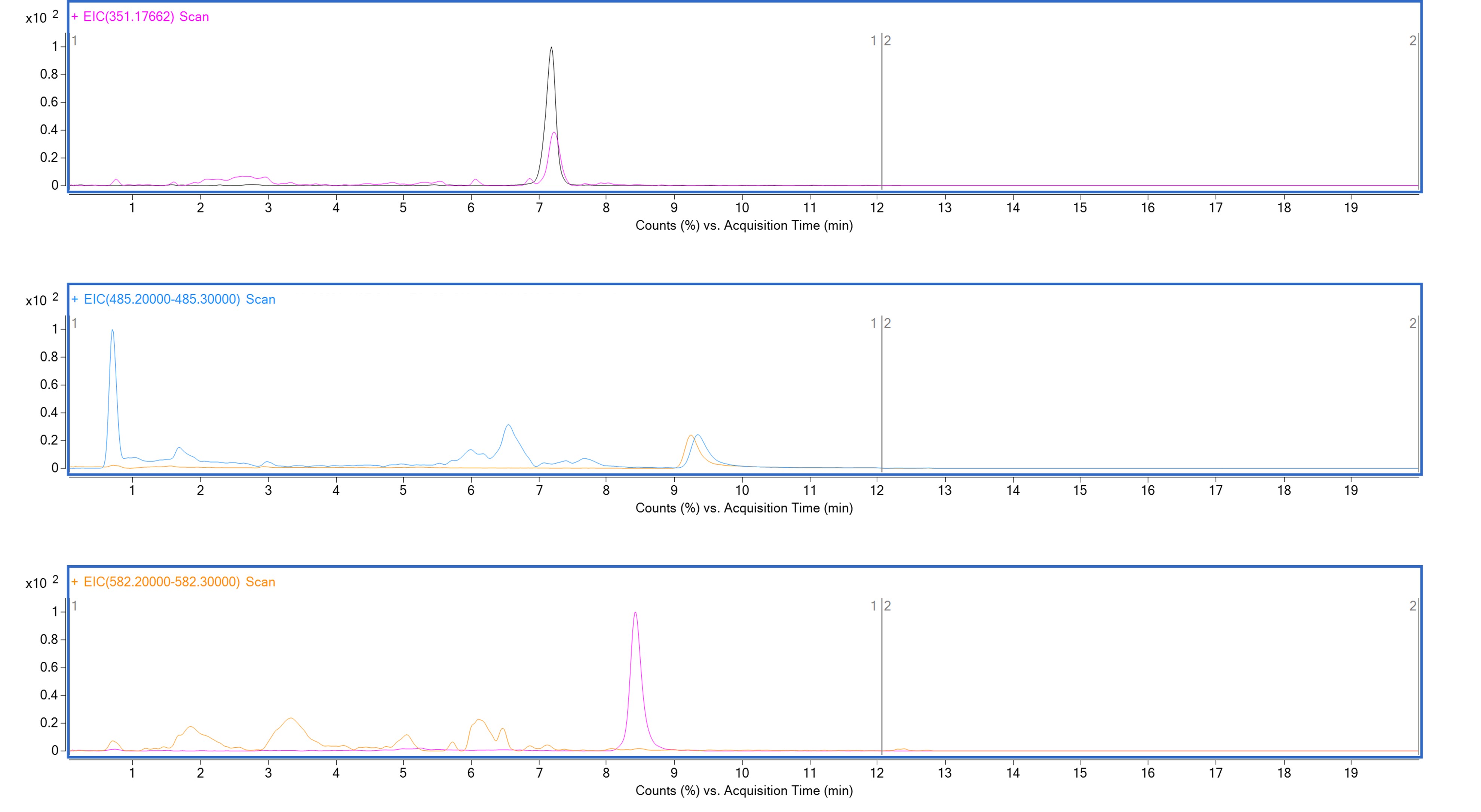


Figure S6. Overlays of extracted ion chromatograms after **Method 6** SPE clean-up of: Spectonomycin (upper) in fresh fermentation medium (pink) and water (black); Kanamycin (middle) in fresh fermentation medium (blue) and water (yellow); Streptomycin (lower) in fresh fermentation medium (yellow) and water (pink)


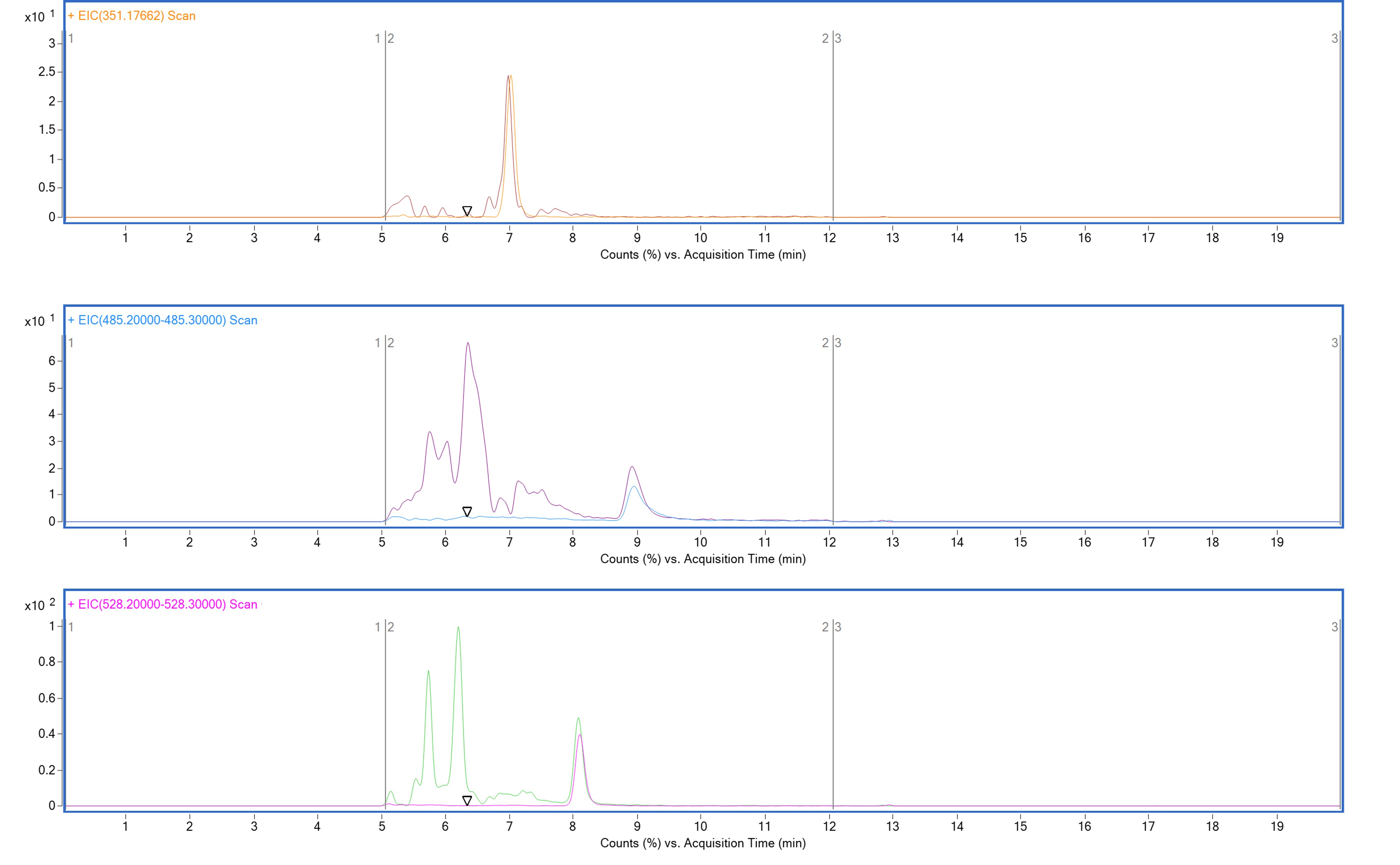


Figure S7. Overlays of extracted ion chromatograms after **Method 5** SPE clean-up of: Spectonomycin (upper) in fresh fermentation medium (brown) and elution solution (yellow); Kanamycin (middle) in fresh fermentation medium (purple) and elution solution (blue); Hygromycin B (lower) in fresh fermentation medium (green) and elution solution (pink)


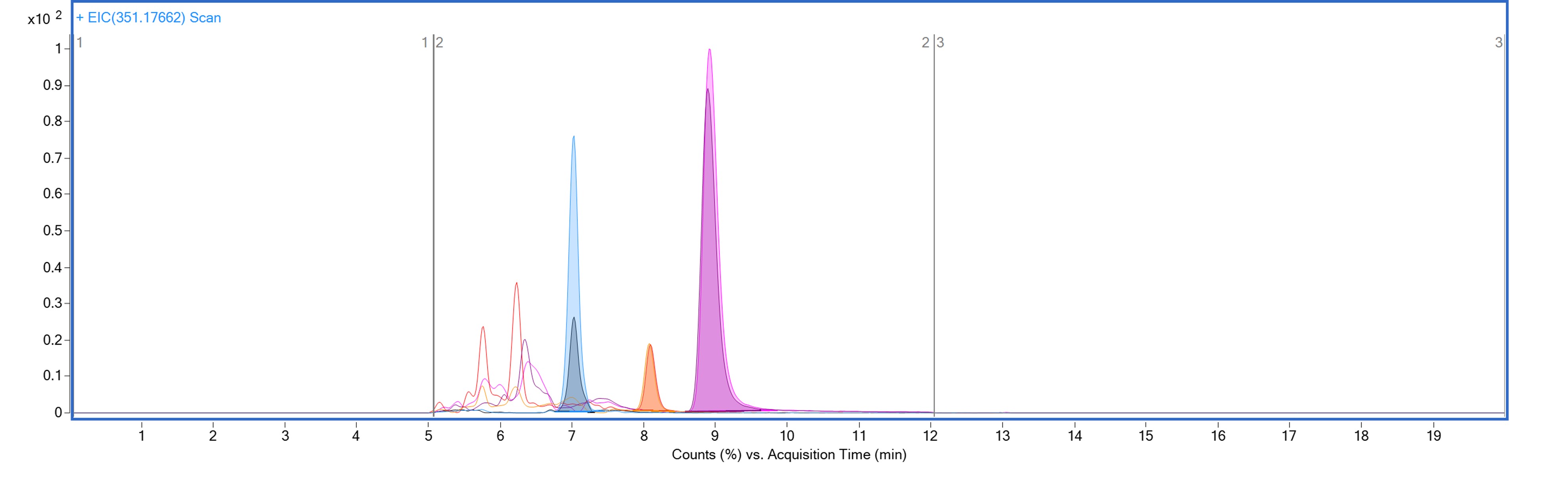


Figure S8. Overlay of extracted ion chromatograms of: Spectinomycin (blue), Hygromycin B (yellow) and Kanamycin (purple) spiked in fresh fermentation medium after SPE and of Spectinomycin (black), Hygromycin B (red) and Kanamycin (pink) spiked in fresh fermentation medium before SPE

1. Liu, Q.; Li, J.; Song, X.; Zhang, M.; Li, E.; Gao, F.; He, L. Simultaneous Determination of Aminoglycoside Antibiotics in Feeds Using High Performance Liquid Chromatography with Evaporative Light Scattering Detection. *RSC Advances*, 2017, *7*, 1251–1259. <https://doi.org/10.1039/C6RA26581B>.
2. Song, S.; Jiang, M.; Yao, J.; Liu, H.; Dai, X.; Wang, G. Alkaline-Thermal Pretreatment of Spectinomycin Mycelial Residues: Insights on Anaerobic Biodegradability and the Fate of Antibiotic Resistance Genes. *Chemosphere* **2020**, *261*, 127821. <https://doi.org/10.1016/j.chemosphere.2020.127821>.
3. Yang, J.; Rainville, P. D. Analysis of Aminoglycosides in Foods Using a Zwitterionic Stationary Phase and Liquid Chromatography-Tandem Mass Spectrometry, 2022.
